# A Comprehensive Assessment of the Effects of *Bt* Cotton on *Coleomegilla maculata* Demonstrates No Detrimental Effects by Cry1Ac and Cry2Ab

**DOI:** 10.1371/journal.pone.0022185

**Published:** 2011-07-12

**Authors:** Yunhe Li, Jörg Romeis, Ping Wang, Yufa Peng, Anthony M. Shelton

**Affiliations:** 1 Department of Entomology, Cornell University/NYSAES, Geneva, New York, United States of America; 2 State Key Laboratory of Plant Disease and Insect Pests, Institute of Plant Protection, Chinese Academy of Agricultural Science, Beijing, China; 3 Agroscope Reckenholz-Tänikon Research Station ART, Zurich, Switzerland; Cairo University, Egypt

## Abstract

The ladybird beetle, *Coleomegilla maculata* (DeGeer), is a common and abundant predator in many cropping systems. Its larvae and adults are predaceous, feeding on aphids, thrips, lepidopteran larvae and plant tissues, such as pollen. Therefore, this species is exposed to insecticidal proteins expressed in insect-resistant, genetically engineered cotton expressing Cry proteins derived from *Bacillus thuringiensis* (*Bt*). A tritrophic bioassay was conduced to evaluate the potential impact of Cry2Ab- and Cry1Ac-expressing cotton on fitness parameters of *C. maculata* using *Bt*-susceptible and -resistant larvae of *Trichoplusia ni* as prey. *Coleomegilla maculata* survival, development time, adult weight and fecundity were not different when they were fed with resistant *T. ni* larvae reared on either *Bt* or control cotton. To ensure that *C. maculata* were not sensitive to the tested Cry toxins independent from the plant background and to add certainty to the hazard assessment, *C. maculata* larvae were fed artificial diet incorporated with Cry2Ab, Cry1Ac or both at >10 times higher concentrations than in cotton tissue. Artificial diet containing E-64 was included as a positive control. No differences were detected in any life-table parameters between Cry protein-containing diet treatments and the control diet. In contrast, larvae of *C. maculata* fed the E-64 could not develop to the pupal stage and the 7-d larval weight was significantly negatively affected. In both feeding assays, the stability and bioactivity of Cry proteins in the food sources were confirmed by ELISA and sensitive-insect bioassays. Our results show that *C. maculata* is not affected by *Bt* cotton and is not sensitive to Cry2Ab and Cry1Ac at concentrations exceeding the levels in *Bt* cotton, thus demonstrating that *Bt* cotton will pose a negligible risk to *C. maculata*. More importantly, this study demonstrates a comprehensive system for assessing the risk of genetically modified plants on non-target organisms.

## Introduction

Cotton is one of the most important economic crops worldwide. In 2006, it was grown in >75 countries with a total production of 27 billion kilograms, and supplied almost 40% of the global demand for fiber [1], [2]. However, many species of insect pests attack cotton plants, and the resulting damage can cause enormous yield losses. Therefore, a substantial part of the cotton production budget is allocated to controlling insect pests. Before the use of insect-resistant genetically engineered (IRGE) cotton, the cotton crop accounted for an estimated 22.5% of the total insecticide used worldwide [1], [2]. Commercial transgenic cotton, expressing one or two Cry toxins derived from the soil bacterium *Bacillus thuringiensis* (*Bt*), has been shown to be effective against many lepidopteran pests including *Heliothis virescens* (Fabricius), *Helicoverpa armigera* (Hübner), *Helicoverpa zea* (Boddie), *Pectinophora gossypiella* (Saunders) and *Trichoplusia ni* (Hübner) in the field and laboratory [3]–[6]. Because of the effectiveness of *Bt* cotton and the resulting significant reduction in the use of broader spectrum insecticides [7], the area grown to *Bt* cotton has increased rapidly around the world [8].

Like any technology there have been questions about the potential risks *Bt* cotton may have on the environment [9]. One of the major ecological concerns regarding the environmental impact of *Bt* plants is their potential effects on non-target organisms since they provide important ecosystem services such as biological control (predators and parasitoids) [10]–[13].

The ladybird beetle *Coleomegilla maculata* (DeGeer) is a common and abundant predator found in many cropping systems worldwide [14]. Both larvae and adults of *C. maculata* are predaceous, feeding on aphids, thrips, and lepidopteran eggs and young larvae [15], [16]. In addition to prey, *C. maculata* also feeds on plant tissues, such as pollen [16], [17]. Therefore *C. maculata* can be directly or indirectly exposed to Cry proteins in several ways when feeding in *Bt* crops. Since *C. maculata* represents an important group of predatory arthropods that is exposed to GE plant-expressed insecticidal proteins in the field and since the species is suitable for laboratory studies, it is commonly used to study the non-target impacts of insecticidal proteins to support the environmental risk assessment of IRGE plants [15], [18], [19].

Previous work on the effects of IRGE plants on *C. maculata* focused on *Bt* maize expressing Cry3Bb1 or Cry1Ab proteins, and a number of studies have revealed that the insect does not appear to be directly negatively affected by these two Cry proteins [15], [16], [18], [20]. However, questions persist about whether effects would be seen if they fed directly on plant tissues or on insects that had fed on plant tissue. Despite the fact that dual *Bt* gene cotton (BollGard II® ) has been planted in several countries [1], [2] and *C. maculata* has the potential to be exposed to cotton-expressing Cry2Ab and Cry1Ac proteins by feeding on prey or cotton pollen in the fields, no studies have been conducted on the potential effects of *Bt* cotton on this species.

In the present study we conducted tritrophic bioassays to investigate the potential effects of BollGard II® cotton on *C. maculata* by using *Bt*-resistant and -susceptible *T. ni* as prey and thereby eliminating any prey-quality mediated effects. Furthermore, we conducted a bioassay in which activated and purified Cry2Ab and Cry1Ac were directly fed to the beetle using a novel Tier-I testing system [21]. This system allowed us to expose the test organisms to toxin concentrations that were much higher than those encountered under field conditions, thus adding further safety to the risk assessment [22].

## Results

### Expression level of Cry1Ac and Cry2Ab in *Bt* cotton tissue

**Figure 1:**
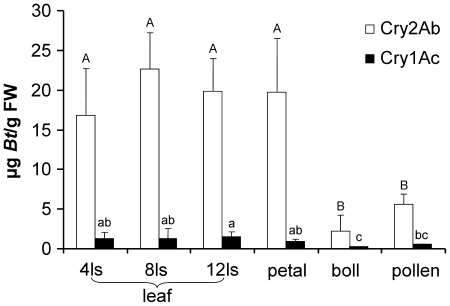
Concentrations (mean+SD) of Cry1Ac and Cry2Ab in *Bt* cotton leaves at different growing stages, petals, bolls and pollen (FW = fresh weight). Different capital letters above open bars indicate significant differences (P<0.05) in the Cry2Ab protein concentrations. Small letters above black bars indicate significant differences (P<0.05) in the Cry1Ac protein concentration. Treatments are significantly different from each other if they do not share the same letter. (N = 3–5; ls = leaf stage).

The *Bt* cotton variety used in the present study was shown to express Cry2Ab at levels ranging from16.8 to 22.7 µg/g fresh weight of cotton leaves and Cry1Ac from 1.3 to 1.5 µg/g fresh weight of cotton leaves, respectively (Fig. 1). No significant difference was detected for Cry1Ac or Cry2Ab in cotton leaves at different growth stages (one-way ANOVA; for both Cry1Ac and Cry2Ab, P>0.40). The expression levels of Cry proteins in petals were similar to those in leaves (P>0.5). Whereas bolls and pollen contained significantly or marginally significantly lower concentrations of the two Cry proteins in comparisons to leaves or petals, there was no significant difference for either Cry protein content in bolls and pollen (P>0.05) (Fig. 1).

### Bioassay with *T. ni*

When susceptible *T. ni* larvae were fed *Bt* cotton leaves, no larva survived the 7 d of the experiment. In contrast, 80.0% of the susceptible *T. ni* larvae survived when fed control leaves. The survival of resistant *T. ni* larvae were 72.4% and 79.3% when fed leaves from *Bt* cotton or control plants, respectively. This difference was not significant (*X* ^2^ = 0.38, P = 0.54). Similarly, % survival did not differ between larvae from the resistant and the susceptible *T. ni* strain when fed non-*Bt* cotton (*X* ^2^ = 0.004, P = 0.95).

### Tritrophic bioassay with *C. maculata*

Larvae of *C. maculata* fed artificial diet spent a significantly shorter time to develop to the adult stage compared to those fed *T. ni* larvae (Mann-Whitney U test; all P<0.0001) (Table 1). Development from neonate to adult took significantly longer when *C. maculata* were fed *Bt* cotton-reared resistant *T. ni* than when fed susceptible larvae reared on control cotton (P<0.0001, adjust α = 0.0083). However, no significant differences were detected for *C. maculata* fed resistant *T. ni* that had been reared on either control or *Bt* cotton (U = 223.0, P = 0.013, adjusted α = 0.0083) or for *C. maculata* that had been fed susceptible or resistant *T. ni* larvae reared on control cotton (U = 221.0, P = 0.018, adjusted α = 0.0083). There were no significant differences detected for any other life-table parameters tested among the four treatments (Table 1).

**Table 1:** Prey-mediated effects on life-table parameters of *Coleomegilla maculata* when fed *Trichoplusia ni* larvae that were reared on Cry1Ac/Cry2Ab-expressing *Bt* cotton leaves or the corresponding non-transformed cotton leaves.

Parameters	Artificial diet	Control cottonSusceptible *T. ni*	Control cottonResistant *T. ni*	*Bt* cottonResistant *T. ni*
Pre-imaginal survival (%)* †	83.3 a	96.4 a	93.1 a	96.3 a
Developmental time to adult (d ± SE)‡	15.3±0.09 c	16.4±0.34 b	16.6±0.12 ab	17.0±0.15 a
Female fresh weight (mg ± SE)§	13.4±0.50 a	11.7±0.53 a	11.8±0.29 a	12.2±0.41 a
Male fresh weight (mg ± SE)§	10.5±0.29 a	10.6±0.33 a	9.75±0.26 a	9.66±0.19 a
Total fecundity (± SE)§	28.6±4.44 a	32.1±7.52 a	29.8±6.00 a	28.8±6.24 a

The experiment started with 30 larvae per treatment.

Means in a row followed by the same letter are not significantly different (P>0.05).

*Pre-imaginal survival = Number of pupae/number of first instar larvae×100.

†Chi-square test with Bonferroni correction (adjusted α = 0.0083).

‡Mann-Whitney *U*-test with Bonferroni correction (adjusted α = 0.0083).

§One-way ANOVA.

**Figure 2:**
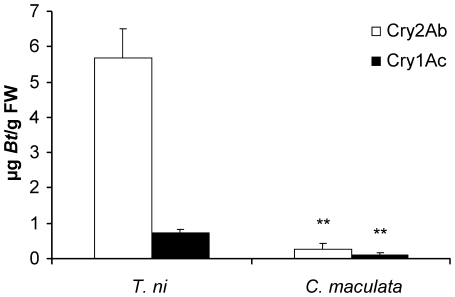
Concentrations (mean+SD) of Cry1Ac and Cry2Ab in larvae of *Trichoplusia ni* and *Coleomegilla maculata* (FW = fresh weight). 2nd instar larvae of *Trichoplusia ni* fed on BollGard II® cotton for 3 days, and the 2nd instar larvae of *Coleomegilla maculata* had fed *Bt*-cotton reared *T. ni* larvae for 3 days (based on fresh weigh). The asterisks “**” indicate the level of significance (P<0.01) in the Cry protein (Cry2Ab or Cry1Ac) concentrations between *T. ni* and *C. maculata*. (N = 5).

The average Cry2Ab concentration in the resistant *T. ni* larvae that had fed on *Bt* cotton leaves for 3 d was 5.68 µg/g fresh weight and the average Cry1Ac concentration was 0.82 µg/g fresh weight of the insects, both figures which were approximately 20–30% of the Cry protein contents in cotton leaves (Figs. 1 and 2). The average Cry toxin concentrations in *C. maculata* larvae were 21-fold lower for Cry2Ab and 6-fold lower for Cry1Ac compared to the concentrations detected in *T. ni* larvae. These differences were highly significant (Cry2Ab: t = 4.91, df = 6.0, P = 0.003; Cry1Ac: t = 4.51, df = 4.0, P = 0.004).

Sensitive-insect bioassays showed that the mortality of *P. xylostella* larvae was 67% after being fed a combination of Cry2Ab and Cry1Ac treated diet for 7 d, which was much higher than the 6.7% mortality that occurred in the control treatment. These results confirm that *C. maculata* was exposed to active toxic proteins in our assay system.

### Direct feeding bioassay with *C. maculata*

**Figure 3:**
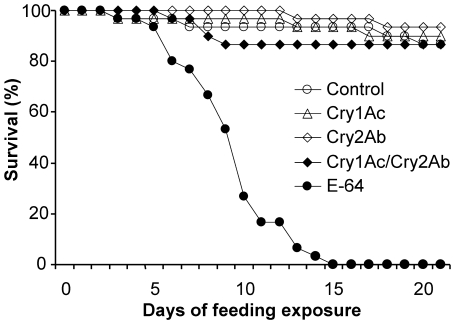
Survival of *Coleomegilla maculata* fed pure artificial diet or diet containing insecticidal protein. Per g dry weight, 100 µg Cry1Ac, 400 µg Cry2Ab, 100 µg Cry1Ac and 400 µg Cry2Ab, or 150 µg E-64 (positive control) were incorporated. Pure diet served as a negative control. (N = 30).

More than 83% of *C. maculata* reached the adult stage in the treatments with Cry proteins and the control treatment, and no significant differences were detected between any Cry toxin treatment and the control (Chi-square test; P>0.05) (Table 2). In contrast, all *C. maculata* died before reaching pupation in the E-64 treatment (E-64 was used here as a positive control since a previous study had shown that it is toxic to *C. maculata* larvae) (Table 2, Fig. 3). Survival analysis showed that there are significant differences among the survival curves of different treatments (*X* ^2^ = 93.7, df = 4, P<0.001). Pairwise comparisons showed that *C. maculata* had a significantly lower survival rate when fed artificial diet incorporated with E-64 compared to those fed the standard artificial diet (P<0.001), and no statistical differences were found between any Cry protein treatment and the control (P>0.30). No differences were found for any Cry protein treatment compared to the control treatment for the following parameters: percentage of larvae developing to adults, developmental time to pupae or adult, larval weight and adult weight (P>0.05) (Table 2). The weight of 7-d old larvae was significantly lower in the E-64 treatment compared to those in the control treatment (Dunnett test; P<0.001) (Table 2).

**Table 2:** Impact of purified Cry1Ac, Cry2Ab and E-64 provided in artificial diet on survival and development of *Coleomegilla maculata*.

Treatment	Larvae developing to adults (%)*	Days to pupation (± SE)†	Days to adult emergence (± SE)†	Seven days larval weight (mg ± SE)‡	Newly emerged adult weight (mg ± SE)‡
Control	86.7	13.4±0.18	16.3±0.21	4.55±0.15	12.2±0.32
Cry1Ac	90.0	13.2±0.15	16.3±0.16	4.99±0.15	12.5±0.30
Cry2Ab	93.3	13.3±0.12	16.2±0.12	4.67±0.17	11.8±0.32
Cry1Ac/Cry2Ab	83.3	13.2±0.15	16.0±0.15	4.57±0.18	12.3±0.35
E-64	0	–	–	0.33±0.02**	–

Larvae of *C. maculata* were fed an artificial diet containing 100 µg Cry1Ac, 400 µg Cry2Ab, 100 µg Cry1Ac and 400 µg Cry2Ab, or 150 µg E-64 (positive control) per g fresh weight of artificial diet. Pure diet served as a negative control (N = 30). The experiment lasted until adult emergence.

Statistical comparisons were made separately for each of the insecticidal proteins with the control. Asterisks denote significant differences:

**P<0.01.

*Chi-square test with Bonferroni correction (adjusted α = 0.017).

†Mann-Whitney U-test with Bonferroni correction (adjusted α = 0.017).

‡Dunnett test.

The extraction efficiency for the Cry2Ab protein in the artificial diet was ca. 62%, with a measured mean (± SE) concentration of 247.6±8.9 µg/g fresh weight of artificial diet. After the 2-d feeding period, the concentration decreased by 10.4% to 222.2±11.6 µg/g fresh weight of artificial diet, and no statistical difference was detected between the treatments (Student's t-test; t = 1.74, df = 4, P = 0.16). For the Cry1Ac protein, 51.3% of the calculated concentration was detected in the diet, with a measured mean (± SE) concentration of 51.3±2.6 µg/g fresh weight of the diet. After 2 d, a significant decrease of 24% to 39.2±2.3 was observed (t = 3.44, df = 4.0, P = 0.026). Sensitive-insect bioassays showed that the mortality of *P. xylostella* larvae was 83% and 67% after being fed Cry2Ab and Cry1Ac protein treated diet for 7 d, respectively, which was much higher than the 10% mortality that occurred in the control treatment.

## Discussion

The risk that an IRGE crop poses for a non-target organism depends on the toxicity of the transgene product to the organism and its probability to be exposed to concentrations that cause adverse effects in the field [12], [23], [24]. Thus, when assessing the effects of *Bt* cotton on non-target organisms, the level of Cry proteins in various cotton tissue is an important factor to determine. Leaves of the *Bt* cotton variety used in the present study expressed Cry2Ab at levels ranging from 16.8 to 22.7 µg/g fresh weight and Cry1Ac at levels ranging from 1.3 to 1.5 µg/g fresh weight. While the petals contained similar amounts of Cry proteins as compared to leaves, the concentrations measured in bolls and pollen were 2–10 times lower. The protein expression pattern and levels detected in our glasshouse-grown *Bt* cotton plants were similar to those previously reported from field-grown plants [25], [26].

Given the high expression levels in leaves, we used larvae of *T. ni* fed with *Bt* cotton leaves to expose *C. maculata* to high doses of the *Bt* Cry proteins. We used a strain of *T. ni* in our study that was highly resistant to Cry2Ab and Cry1Ac as demonstrated by the similar survival after 7 d of feeding on *Bt* cotton or control cotton leaves. By using resistant larvae as prey for *C. maculata*, possible indirect effects on the predator that could be due to reduced nutritional quality of prey after ingestion of the insecticidal proteins were minimized. Such prey-quality mediated effects have often been observed in tritrophic studies [11], [27] and the use of resistant strains of pests as prey has been suggested as a way to test the direct toxic effects of the plant-expressed insecticidal compounds [22], [28]–[30].

The tritrophic experiment confirmed that that *T. ni* is a suitable prey for *C. maculata* [31]. With the exception of a slightly shorter development time, all the tested life-table parameters were similar for *C. maculata* when fed *T. ni* compared to those fed the artificial diet. The experiments did not reveal any adverse effects on the fitness of *C. maculata* after ingestion of *Bt*-resistant *T. ni* larvae that were fed *Bt* cotton leaves compared with those that were fed the corresponding non-transformed cotton leaves. Interestingly, *C. maculata* larvae fed susceptible *T. ni* larvae reared on control cotton required a significantly shorter time to develop to adults compared with those fed the resistant *T. ni* from *Bt* cotton. The results suggest that the difference was not due to the Cry toxins, but may be caused by the different nutrient composition in *Bt*-resistant and susceptible *T. ni* larvae due to their different genetic backgrounds or due to the interactions of their genetic backgrounds and food sources. A recent study reported changes in sugar concentration and composition in larvae of *Helicoverpa armigera* (Hübner) of a Cry1Ac-susceptible strain and a reduced glycogen content in larvae of a Cry1Ac-resistant strain when fed *Bt* (Cry1Ac) cotton [30].

In the present study, ELISA measurements showed that *Bt* cotton-reared *T. ni* contained high concentrations of Cry2Ab (ca. one third the level in cotton leaves) and Cry1Ac (ca. half of the level in cotton leaves). In addition, Cry proteins were detected in *C. maculata* when fed *Bt*-cotton reared *T. ni* larvae, although the *Bt* protein levels were 21 times lower for Cry2Ab and 6 times lower for Cry1Ac compared to the concentrations in the prey. Such a strong dilution of toxins has been reported from other tritrophic experiments in which prey fed on *Bt* plants was used to expose predators to the insecticidal compounds. For example, Li and Romeis [32] had fed ladybird beetles, *Stethorus punctillum* (Weise), with spider mites, *Tetranychus urticae* (Koch), that had been reared on Cry3Bb1-expressing *Bt* maize. The ELISA results revealed Cry protein concentrations in the ladybird beetle larvae and adults that were 6 times and 20 times lower, respectively, than that measured in the spider mite prey. Similarly, first and second instars of larvae of *Adalia bipunctata* contained toxin concentrations that were 7–12 times lower than that measured in spider mites that had fed Cry1Ab or Cry3Bb1-expressing maize [33]. One possible reason for the low Cry protein concentrations measured in the predators is the fact that they excrete the proteins, as was previously reported for snowdrop lectin (*Galantus nivalis* agglutinin) and larvae of two ladybird beetle species [34].

Sensitive insect bioassays that were carried out with *P. xylostella*, confirmed the biological activity of the Cry proteins after ingestion by *T. ni* larvae. Thus, these data suggested that *C. maculata* were exposed to high levels of biologically active Cry2Ab and Cry1Ac proteins throughout the duration of the tritrophic feeding assays but are not sensitive to these proteins in their prey.

To draw a general conclusion about the sensitivity of *C. maculata* to Cry2Ab or Cry1Ac independent from the plant background, and to increase the exposure level of *C. maculata* to the test proteins, a direct feeding bioassay using activated and purified toxic proteins incorporated into artificial diet, was carried out. A concentration that was >10 times higher than that measured in cotton leaves was used to achieve a worst-case exposure scenario and add a safety margin and thus increase the certainty of the conclusions of the hazard assessment [12], [22], [24]. Similar to the tritrophic experiment using *T. ni* larvae as prey, no adverse impact of the two Cry toxins was found on *C. maculata* for any of the test life-table parameters tested. In this bioassay, the larvae fed E-64 incorporated diet could not develop to adults, and the 7 d old larval weights were substantially lower than those fed untreated diet or Cry toxin-incorporated diet. This positive control demonstrated that our experimental system was able to detect adverse effects caused by toxic substances in the diet. Although the ELISA measurements showed a significant degradation of the Cry proteins in the artificial diet, the concentration measured after the 2 d feeding exposure was still more than 10 times higher than that measured in *Bt* cotton leaves. Furthermore, bioactivity of both purified proteins was confirmed by sensitive-insect bioassays. Therefore, the results add confidence to the conclusions drawn from the tritrophic experiment that *C. maculata* is not sensitive to Cry2Ab or Cry1Ac at concentrations that are much higher compared to the concentrations that they may encounter in *Bt* cotton fields. We therefore conclude that *C. maculata* is unlikely to be harmed by the growing of Cry2Ab and Cry1Ac-expressing *Bt* cotton.

In summary, this comprehensive study using a tritrophic bioassay and a direct feeding bioassay provides the most complete information so far on the potential direct and indirect effects of Cry2Ab and Cry1Ac on the ladybird beetle, *C. maculata*, a common and abundant predator in many cropping systems. ELISA measurements and sensitive-insect bioassays confirmed that the test insects were exposed to high concentrations of biologically active Cry proteins throughout the duration of the bioassays. This adds further certainty to the conclusion that *C. maculata* is unlikely to be harmed by the growing of Cry2Ab and Cry1Ac-expressing *Bt* cotton. More importantly the current study provides a critical method for assessing non-target impacts of genetically modified crops that can be useful for further studies in risk assessment of GE crops.

## Materials and Methods

### Insects

A laboratory colony of *T. ni* that had never been exposed to *Bt* [35] was used as a susceptible strain. As a resistant *T. ni* strain, GLEN-BGII was used. This strain is resistant to both the *Bt* toxins Cry1Ac and Cry2Ab and can survive well on BollGard II® cotton plants.

A susceptible strain of *Plutella xylostella* L. (Geneva 88) was used to confirm the bioactivity of the Cry proteins in plant and insect samples. This strain has been maintained on a wheat germ-casein artificial diet for over 250 generations in the laboratory [36].

Larvae of *C. maculata* were collected from a long-term laboratory colony that originated from Pioneer Hi-Bred International, Inc. (Johnston, IA). The insects were maintained in a climatic chamber at 27±1°C, 65±5% RH and 16∶8 L∶D at Cornell University's Department of Entomology at Geneva, NY. Both larvae and adults were reared on decapsulated eggs of brine shrimp, *Artemia franciscana* (Brine Shrimp Direct, Ogden UT) and 1% agar solution provided separately as a water source, a technique which Pioneer has utilized as a diet for *C. maculata*.

### Plants

Seeds of *Bt*-cotton BollGard II® which carries the genes coding for Cry1Ac and Cry2Ab, and the corresponding non-transformed near isoline Stoneville 474, were obtained from Monsanto Company (St. Louis, MO). The two cotton varieties were grown simultaneously in the same growth chambers. Plants were individually grown in 6 L plastic pots filled with Cornell mix soil [37].

### Expression of Cry1Ac and Cry2Ab in cotton tissue

When the cotton plants reached the 4, 8 and 12-leaf stage (ls), five leaf samples (leaf discs of 5 mm in diameter) were collected from five different cotton plants. Each sample was approximately 30 mg and obtained from a middle-upper leaf of a *Bt* or control plant. When cotton plants reached the flowering and boll stages, three to five samples of the petal, pollen, and young boll were separately collected from both *Bt* and control plants. All samples were weighed and put into 1.5-ml centrifuge tubes respectively, and kept at −20°C for Cry protein measurement using ELISA.

### Purified proteins

Cry1Ac was prepared from *B. thuringiensis* strain HD-73 as described by Kain et al. [35]. The bioactivity of Cry1Ac was confirmed in an insect bioassay using neonate *T. ni* larvae. Toxin solutions were sprinkled on the surface of artificial diet and fed to neonate larvae for 7 d. A 60 ng Cry1Ac/cm^2^ diet resulted in 70% larval mortality. Cry2Ab toxin used in this study was provided by the Monsanto Company. Insect bioassays using *H. zea* larvae conducted at Monsanto Company showed that the LC_50_ (concentration resulting in 50% mortality) of our Cry2Ab protein batch was 0.25 µg/ml diet when neonate larvae were fed with protein containing artificial diet for 1 wk. Protease inhibitor E-64 (N-[N-(L-3-trans-carboxyoxirane-2-carbony1)-L-leucyl]-agmatine) was purchased from Roche Biochem (Indianopolis, IN). E-64 was used as a positive control treatment since a previous study had shown that it is toxic to *C. maculata* larvae [21].

### Bioassay conditions

All bioassays were conducted in an environmental chamber at 27±1°C, 65±5% RH and 16∶8 L∶D at Cornell University's Department of Entomology at Geneva, NY.

### Bioassay with *T. ni*

A bioassay was conducted to determine the effects of *Bt* cotton on survival of *T. ni* larvae. Neonates of the resistant or susceptible *T. ni* strain were individually fed with leaf disks either collected from *Bt* cotton or control cotton plants at the 8–10 leaf stage. The leaf disks were placed in 30-ml plastic cups with ventilated lids. In addition, a water-saturated cotton ball was provided on the bottom of each cup to increase humidity. Thirty larvae were tested in each of four treatments: 1) Susceptible *T. ni*+Control cotton (S+C); 2) Susceptible *T. ni*+*Bt* cotton (S+*Bt*); 3) Resistant *T. ni*+Control cotton (R+C); 4) Resistant *T. ni*+*Bt* cotton (R+*Bt*). Cotton leaves were replaced every 2 d. After 7 d of feeding, the number of surviving larvae was recorded.

### Tritrophic bioassay with *C. maculata*

In this experiment, *C. maculata* were fed one of the following four diet treatments: 1) susceptible *T. ni* larvae reared on control cotton leaves (S+C); 2) resistant *T. ni* larvae reared on control cotton leaves (R+C); 3) resistant *T. ni* larvae reared on *Bt* cotton leaves (R+*Bt*), and; 4) brine shrimp eggs and 1% agar solution (artificial diet). To prepare the prey larvae, neonate *T. ni* from the susceptible and resistant strains were reared on control or *Bt* cotton leaves of plants in the 8–10 leaf stage. When *T. ni* larvae reached the 2nd-3rd instar (3–4 d), they were fed *ad libitum* to 2nd instar *C. maculata* together with the corresponding cotton leaf discs in 30-ml plastic cups with ventilated lids. The *C. maculata* larvae had previously been fed with the shrimp eggs-based artificial diet for colony maintenance. The prey was replaced every 2–3 d. The artificial diet has been used for *C. maculata* colony maintenance in our laboratory and is considered to be a suitable food for development and reproduction of *C. maculata*. It was used here as a reference control to assess the suitability of *T. ni* larvae as food for *C. maculata*. The decapsulated shrimp eggs were sprinkled into the plastic cups, and a 1% molten agar/water solid, contained in a 1.5 ml centrifuge tube, was added to the cups. The artificial diet was changed every 2 d. Larvae of *C. maculata* were observed daily and their survival and development were recorded. When the adults emerged, they were sexed and weighed. A male and a female from the same treatment were kept in a Petri dish and allowed to mate. All pairs of *C. maculata* from any treatment were fed shrimp eggs and agar solution for 2 wk and the total number of eggs produced by each female was recorded. The experiment was initiated with 30 *C. maculata* larvae per treatment. For the assessment of the adult beetles, in total between 9 and 12 pairs were observed per treatment.

To document the bioactivity and the movement of Cry proteins through the food chain, *Bt* cotton, 2nd instar *T. ni* and 2nd instar *C. maculata* were kept in a cage with fine mesh screens. Six *Bt* or control cotton plants (10 leaf-stage without flowers) were put into the cage and 3 d later three samples of *T. ni* and *C. maculata* were collected from the cages. The samples were kept at −20°C for subsequent ELISA measurements and sensitive-insect bioassays.

### Direct feeding bioassay with *C. maculata*

To increase certainty of the hazard assessment of *Bt* plants, a Tier-I testing system was developed, in which *C. maculata* could be exposed to concentrations of test compounds in an artificial diet [21] that are higher than those found in plant or prey material. Here, the experimental system was used to assess the toxicity of Cry1Ac and Cry2Ab on *C. maculata*. Five diet treatments were tested: 1) artificial diet (negative control); 2) artificial diet containing Cry1Ac at 100 µg/g fresh weight of diet; 3) artificial diet containing Cry2Ab at 400 µg/g fresh weight of diet; 4) artificial diet containing Cry1Ac at 100 µg/g and Cry2Ab at 400 µg/g fresh weight of diet; 5) artificial diet containing E-64 at 150 µg/g fresh weight of diet (positive control). Diets were replaced every 2 d. The experiment was conducted with 30 neonate *C. maculata* larvae per treatment. The insects were observed daily and their survival and development were recorded until adult emergence. In addition, larval weight was recorded after 7 d. Adult fresh weight was measured within 12 h after emergence.

To ensure the stability and bioactivity of the Cry protein during the 2 d feeding exposure, three samples were collected from each diet prior to feeding the insects and from each diet that has been exposed to the insects for 2 d. The Cry toxin concentrations and the bioactivities in the samples were subsequently determined by ELISA and sensitive-insect bioassays (see below).

### ELISA measurement

The concentrations of Cry2Ab and Cry1Ac in cotton tissue, insects and artificial diet were measured by double-antibody sandwich enzyme-linked immunosorbent assays (DAS-ELISA) using the Cry1Ac and Cry2Ab detection kits from Agdia (Elkhard Indiana, USA). Prior to analysis, all insects were washed in phosphate buffered saline with Tween-20 (PBST) buffer (provided in the kit) to remove any *Bt* toxin from their outer surface.

After adding PBST to the samples at a ratio of at least 1∶10 (mg sample∶µl buffer) in 1.5 ml centrifuge tubes, the samples were fully ground by hand using a plastic pestle. After centrifugation and appropriate dilution of the supernatants, ELISA was performed according to the manufacturer's instructions. The measured OD values were calibrated to a range of concentrations of Cry2Ab and Cry1Ac made from purified toxin solution.

### Sensitive-insect bioassay

Samples of *T. ni* larvae fed *Bt* or non-*Bt* cotton leaves for 3 d from the tritrophic bioassay and samples of artificial diets containing Cry2Ab or Cry1Ac or no protein that had been freshly prepared or had been exposed to insects for 2 d in the direct feeding bioassay were ground using a mortar and a pestle. After centrifugation, the supernatants were collected and the protein concentrations in the extracts were measured by ELISA. Subsequently the extract solutions were appropriately diluted and spread on the surface of an artificial diet for *P. xylostella* [36] resulting in ca. 100 ng Cry toxin on each cm^2^ diet. After 2 h of air-drying, 30 *Bt*-susceptible neonate larvae of *P. xylostella* were distributed onto the diet for each treatment. After 7 d, the mortalities of the insects were recorded. Three replicates were tested in each treatment.

### Statistical analysis

The mean concentrations of Cry proteins in different cotton tissues or artificial diets were compared among the treatments using one-way ANOVA followed by Tukey HSD tests. *Trichoplusia ni* larval survival on *Bt* and non-*Bt* cotton plants was compared using Chi-square tests. In the tritrophic experiment, the treatments were compared with each other. Chi-square tests were performed for the percentage data of pre-imaginal survival and Mann-Whitney U-tests were carried out for the data sets of insect development time (from neonate to pupae or adult), since the assumptions for parametric analyses were not fulfilled. Bonferroni correction was applied to correct for 6 pair-wise comparisons leading to an adjusted α = 0.0083. Data on adult weight and female fecundity were analyzed using one-way ANOVA followed by Tukey HSD tests when significant differences were detected. The Cry protein (Cry2Ab or Cry1Ac) concentrations in *T. ni* and *C. maculata* were compared using Student's t-tests.

For the direct feeding bioassay, comparisons were conducted for each toxin treatment with the untreated control. Percentage data of larvae developing into adults were analyzed using Chi-square tests and Mann-Whitney U-tests were used for the data of insect development time (from neonate to pupae or adult). Bonferroni correction was applied to correct for 3 pair-wise comparisons leading to an adjusted α = 0.017. Larval weight at 7 d and adult weight were analysed using Dunnett tests. The survival response of *C. maculata* to different dietary treatments in direct feeding experiment was analyzed using the Kaplan-Meier procedure and Logrank test. The Cry protein concentrations in artificial diets were compared using Student's t-tests.
